# Supplementary material for: Divergence of TORC1-mediated Stress Response Leads to Novel Acquired Stress Resistance in a Pathogenic Yeast
Source: bioRxiv. 2023 Sep 19:2023.06.20.545716. Preprint. [Version 2] doi: 10.1101/2023.06.20.545716 (PMC10541095; doi:10.1101/2023.06.20.545716)
Supplement: Supplement 2 [file media-2.docx]

# Supplementary Text S1 Predicting TF binding sites in *C. glabrata* *CTA1* promoter

## *CgCTA1* 1000bp upstream sequence

5’ -
AGCTGGTCGTTCAACTGAGAAAGTTCCAGCTTCTAAGCTATTATTTTGTCATCAAGCCTG
TGATCCCAATCTCTTTTGAGATTATCACTACTAGTTAAACACTTGTCGGAGATTAGAAGT
TTTCTCTTTCACAGGAGATATCATCTCACCAACAAAATGCTGCCTTCATCCTCTCTCTAC
CCATGTTCCTAACCTATTTGAGTTCTCTCGTTCTGTTTTGGTTCTGGGCCATGCCACAGT
ACTCTTAGTAATTGCCCTTTATCCTACTGGCTCTTGTAACCCTTCTTAACCCTTTACTAG
CTATTGCTCAAGGATAGCACAAAGGGCCATGGGATGGCTAATTCCTTAACCTTTAAAATT
TAGGGAGCCTTCCAGTTTCTCTTGTTGAATAACGGAGGTTTGTATGGAGGGAAAACGCTA
ATTTCGATGAAGGAGAGATGCCCTTCGCAAATGTGTCGAAGTTTCTCACTATTCCTTCAA
CCCTCTTTCCTCCTTTTCCTTCGGCAATAGACACTGCACTATATTTGGAGTAAAATGTCC
GTCTTTCATCGTTAAACTACCTTGAGAGAACTTAAAGAAAACTCCAACTAATTACCTTGG
AACTTGGGATAAAGAAATTAGTAACCCCTCATGCTAGTTCTCTGAAAAGTATCTGAAAAT
TTACTGTTTGCAACAATAAGTTATGGATGCAATTACAAATTGTGCTTAAGACCTAGACTA
AATTAATAACAATTATTATTTCCCTTAAGTCCTTTCTCAATAATCAATAGTTTGGATAGC
TATATAAAGGGTAAGCCTTTACCATCATCAAGCCTTGGAACACATCCTTTATCCTTTGTT
GTTCTTATTGTAAAATATATAAATACAACAACAAACCAAATCTTGAAAGCATCAATTATA
TACATAATAGATACTTTCAATCAACAAAAAATCAACATTATAACGCTAATTTTTTGTTCC
TTTTGGCTTATTAGATAACTTCCCACAATTGAAAAAAAAC

- 3’ (followed by the start codon ATG)

## Prediction result

| **TF** | **Hit** | **% max Score** | **Position (from 3’)** | **Motif** |
| --- | --- | --- | --- | --- |
| Msn4 | CCCCT | 100 | 372 - 376 | Msn4 #518 |
| Skn7 | GGCCAT | 91.9 | 769 - 774 | Skn7 #583 |
| Skn7 | GGCCAT | 91.1 | 676 - 671 | Skn7 #583 |

## Position frequency matrices (PFMs) used for prediction

Msn4 #518

A 0.010683761 0.002136752 0.002136752 0.002136752 0.002136752

T 0.002136752 0.002136752 0.002136752 0.002136752 0.831196581

G 0.002136752 0.002136752 0.002136752 0.002136752 0.002136752

C 0.985042735 0.993589744 0.993589744 0.993589744 0.164529915

Skn7 #583

A 0.000961538 0.000961538 0.000961538 0.000961538 0.485576923 0.208653846

T 0.000961538 0.000961538 0.000961538 0.000961538 0.000961538 0.320192308

G 0.997115385 0.997115385 0.000961538 0.1625 0.366346154 0.293269231

C 0.000961538 0.000961538 0.997115385 0.835576923 0.147115385 0.177884615

Skn7 #380 (no hit found with this motif)

A 0.054 0.084 0.032 0.054 0.202 0.035 0.101 0.022 0.108

T 0.059 0.217 0.052 0.324 0.121 0.017 0.046 0.035 0.098

G 0.84 0.279 0.042 0.309 0.363 0.876 0.428 0.03 0.2

C 0.048 0.42 0.874 0.313 0.314 0.072 0.425 0.913 0.593
