# Supplementary material for: Divergence of TORC1-mediated Stress Response Leads to Novel Acquired Stress Resistance in a Pathogenic Yeast
Source: bioRxiv. 2023 Sep 19:2023.06.20.545716. Preprint. [Version 2] doi: 10.1101/2023.06.20.545716 (PMC10541095; doi:10.1101/2023.06.20.545716)
Supplement: 1 [file NIHPP2023.06.20.545716V2-supplement-1.pdf]

bioRxiv preprint doi: https://doi.org/10.1101/2023.06.20.545716; this version posted September 19, 2023. The copyright holder for this preprint (which was not certified by peer review) is the author/funder, who has granted bioRxiv a license to display the preprint in perpetuity. It is made available under aCC-BY 4.0 International license.

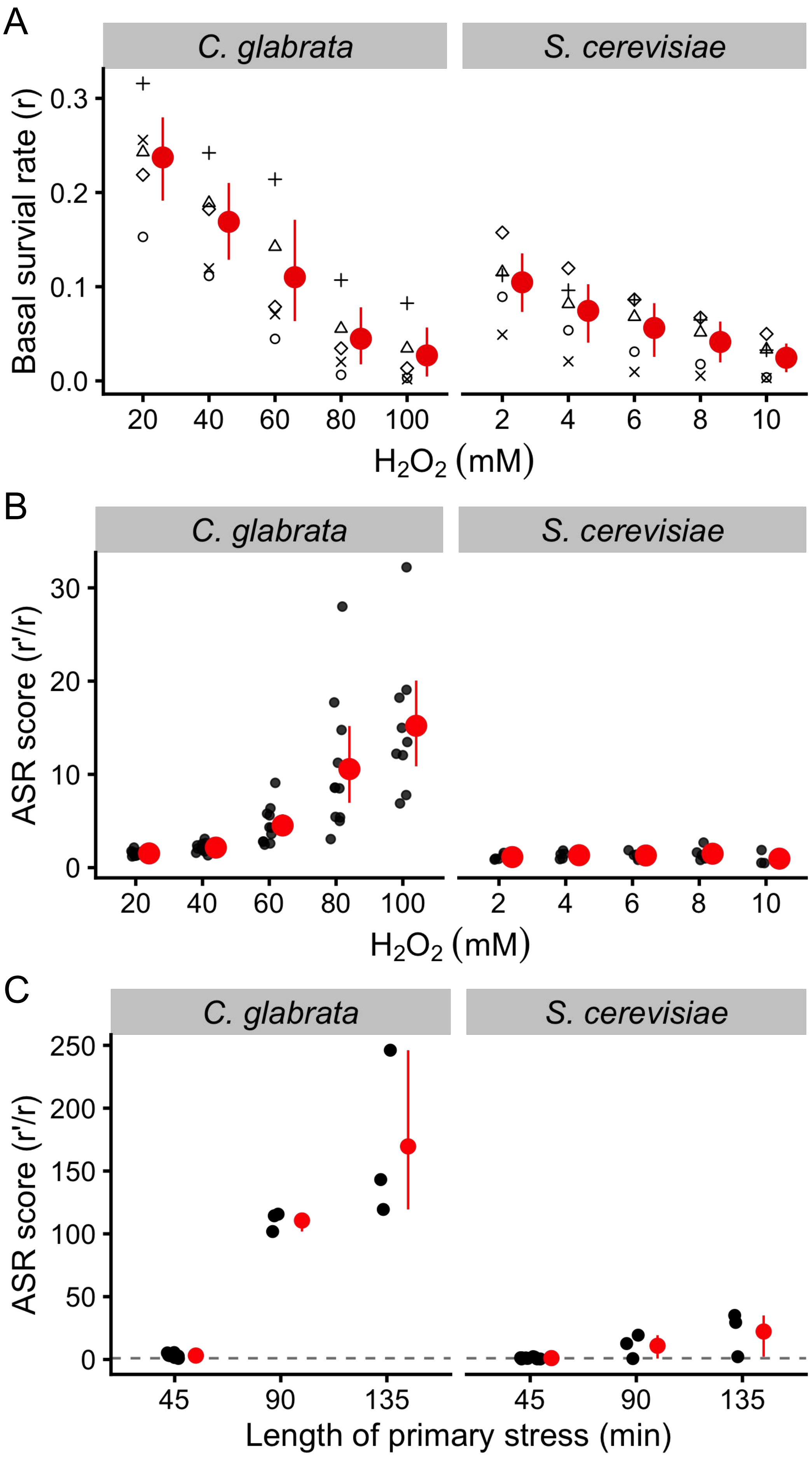

bioRxiv preprint doi: https://doi.org/10.1101/2023.06.20.545716; this version posted September 19, 2023. The copyright holder for this preprint (which was not certified by peer review) is the author/funder, who has granted bioRxiv a license to display the preprint in perpetuity. It is made available under aCC-BY 4.0 International license.

Supplementary Figure 1. Basal survival rates and phosphate starvation induced acquired resistance for H_2O_2 at different primary and secondary stress conditions. (A) Basal survival rates (r) at different H_2O_2 concentrations in *C. glabrata* and *S. cerevisiae* were quantified using Colony Forming Unit (CFU) ratios between cells treated with H_2O_2 and mock treated ones. The red dots and vertical lines show the mean and 95% confidence intervals based on 1000 bootstrap replicates. Individual data points are shown in different shapes grouped by the date of the experiment. (B) Acquired Stress Resistance (ASR) at different H_2O_2 secondary stress levels in the two species. ASR-score is defined as the fold increase in survival after the H_2O_2 treatment as a result of the primary stress (phosphate starvation). r' is the survival rate with the primary stress and r is the same as in (A), i.e., without the primary stress. The red dots and lines have the same meanings as in (A). (C) ASR for H_2O_2 at different primary stress length. 100 mM and 10 mM of H_2O_2 were used as the secondary stress for the two species as in Fig. 1. The dotted line shows an ASR score of 1 (i.e., no increase in survival due to the primary stress). A paired t-test was performed on the underlying survival rates (r and r') for each of the six species-by-duration combinations. After Bonferroni correction, all three tests in *C. glabrata* had $P < 0.05$, while all three tests in *S. cerevisiae* yielded $P > 0.5$

bioRxiv preprint doi: https://doi.org/10.1101/2023.06.20.545716; this version posted September 19, 2023. The copyright holder for this preprint (which was not certified by peer review) is the author/funder, who has granted bioRxiv a license to display the preprint in perpetuity. It is made available under aCC-BY 4.0 International license.

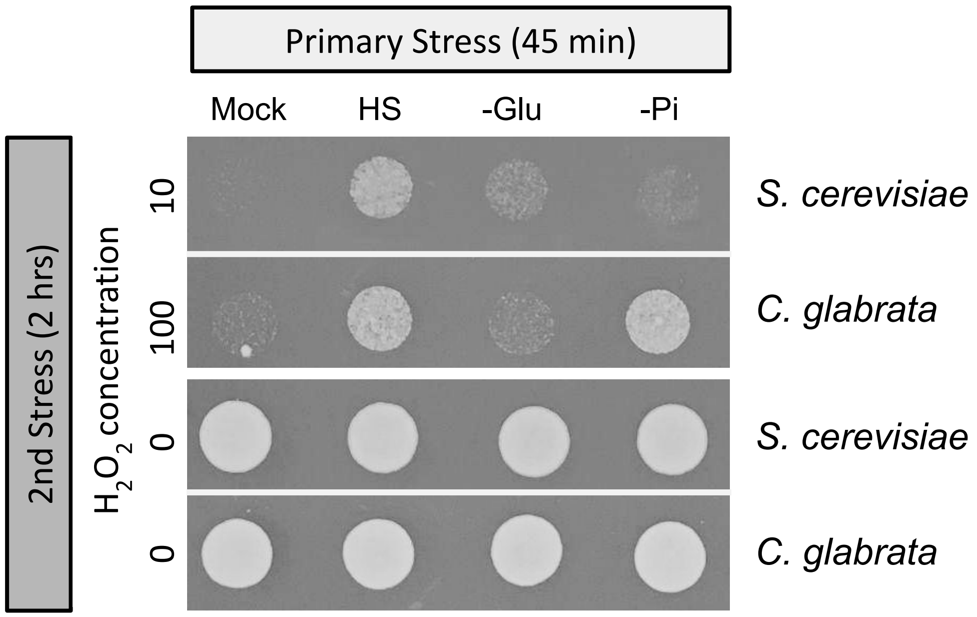

Supplementary Figure 2. Phosphate starvation-induced ASR for H_2O_2 diverge between species while other primary stresses show similar effects. ASR experiment was performed as in Figure 1, with the exception of using different primary stresses as indicated on the top: Mock - rich SC medium; HS - Heat shock at 43C; -Glu - 0.05% glucose (as opposed to 2% in SC); -Pi - no phosphate SC medium. Images were taken 14 hrs and 21 hrs post spotting for *C. glabrata* and *S. cerevisiae* respectively.

bioRxiv preprint doi: https://doi.org/10.1101/2023.06.20.545716; this version posted September 19, 2023. The copyright holder for this preprint (which was not certified by peer review) is the author/funder, who has granted bioRxiv a license to display the preprint in perpetuity. It is made available under aCC-BY 4.0 International license.

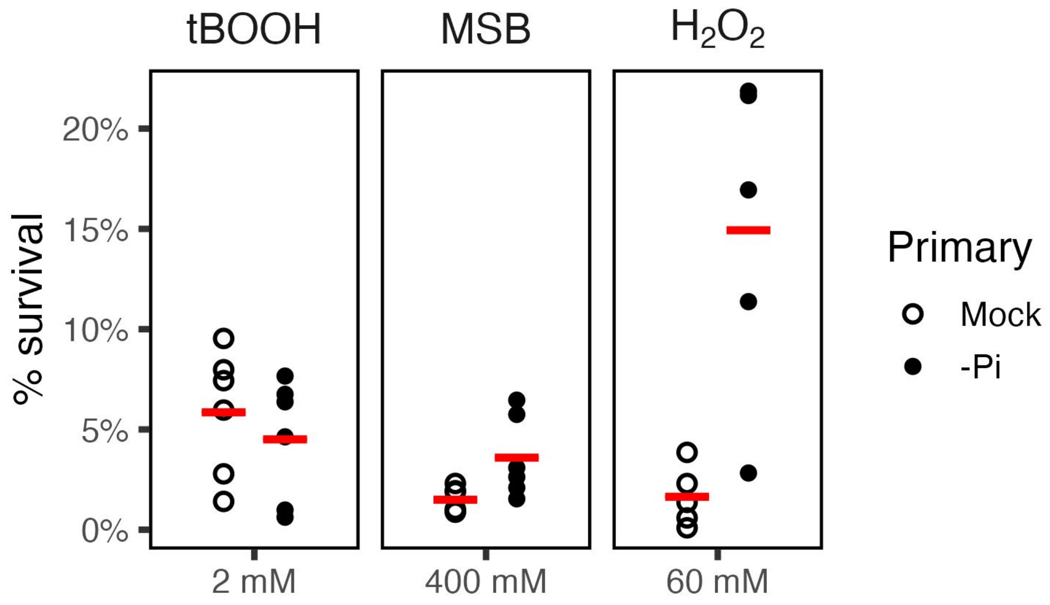

Supplementary Figure 3. Phosphate starvation's ASR effect depends on the type of ROS. We tested the ability of phosphate starvation to provide acquired resistance for two additional ROS, i.e., tert-butyl hydroperoxide and menadione sodium bisulfate (MSB), and compared them to H_2O_2, each at the indicated concentration. No ASR effect was observed for tBOOH (mean ASR-score = 0.71, 95% CI [0.49, 0.88], *P* = 1). There is moderate ASR for MSB (mean ASR-score = 2.41, 95% CI [1.79, 3.00], raw *P* = 0.016). ASR for H_2O_2 is the strongest (mean ASR-score = 16.4, 95% CI [7.7, 25.3], raw *P* = 0.031).

bioRxiv preprint doi: https://doi.org/10.1101/2023.06.20.545716; this version posted September 19, 2023. The copyright holder for this preprint (which was not certified by peer review) is the author/funder, who has granted bioRxiv a license to display the preprint in perpetuity. It is made available under aCC-BY 4.0 International license.

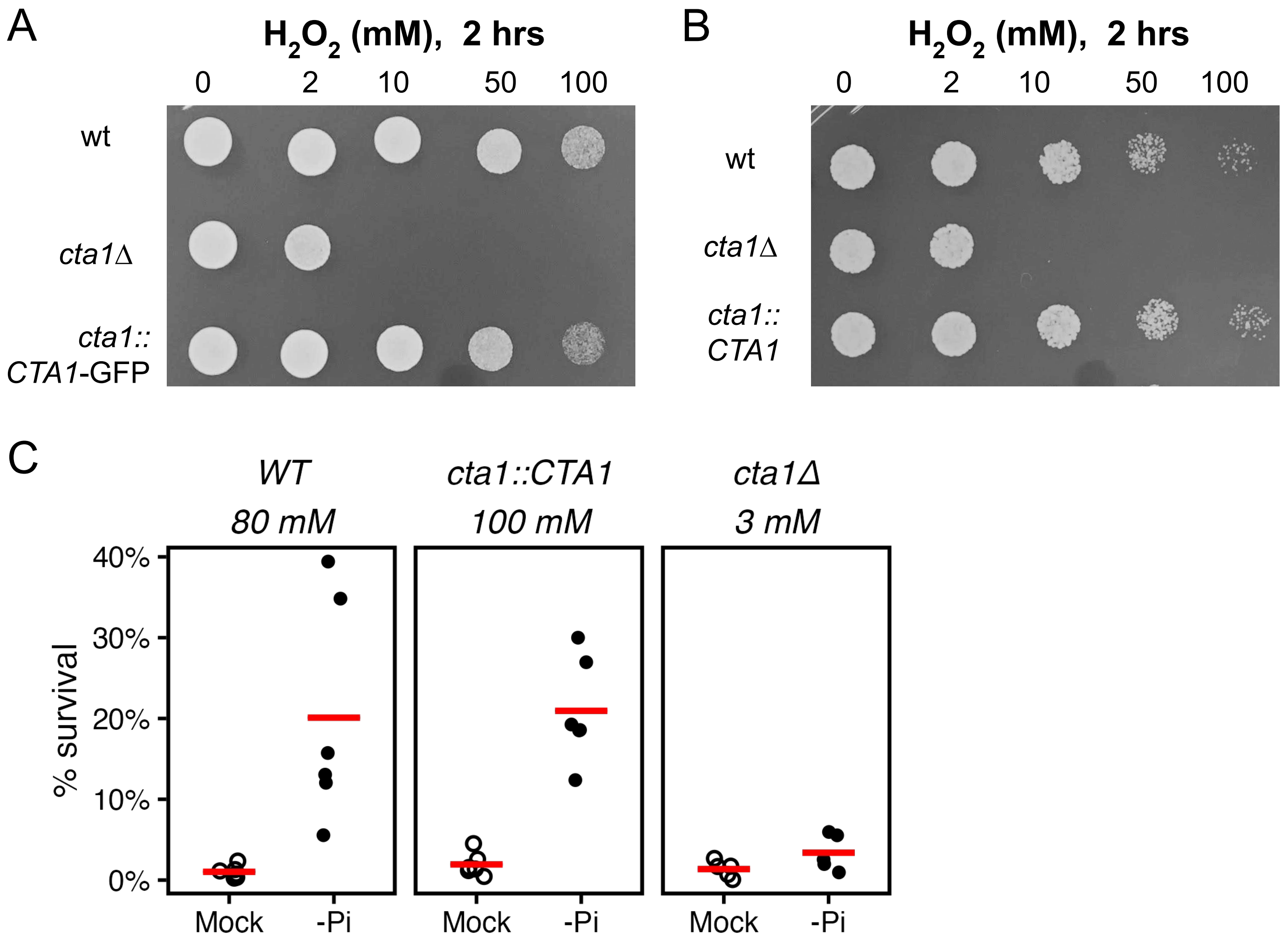

Supplementary Figure 4. *CTA1* complement strain rescues basal H_2O_2 survival and ASR defects in *cta1*Δ. Putting either *CTA1*-GFP (A) or the untagged *CTA1* (B) back to the endogenous locus in the *cta1*Δ background restored the resistance to H_2O_2 compared with the wild type strain. Each strain was treated at the indicated concentrations of H_2O_2 for 2 hours, then spotted onto YPD plates and incubated at 30°C for 48 hours (A) and 20hrs (B). (C) ASR in the wild type, *CTA1* complement (*cta1::CTA1*) and *cta1*Δ strains. The experiment was conducted similarly as in Fig. 3 for wild type and *cta1*Δ strains. Concentrations of H_2O_2 were calibrated to achieve a similar basal survival rates (open circles, Kruskal-Wallis rank sum test for differences among the three groups P = 0.38). ASR-scores for the three strains are (with 95% CI and Wilcoxon signed-rank test P-values in the parenthesis): wild type 23.9 ([14.9, 36.5], P=0.048); *cta1::CTA1* 15.4 ([9.5, 21.3], P=0.048); *cta1*Δ 5.1 ([2.0, 10.6], P=0.093). The difference in ASR-score is significant between *cta1*Δ and wild type, but not between *cta1::CTA1* and wild type (Mann-Whitney U test P = 0.034 and 0.48, respectively, Bonferroni-corrected).

bioRxiv preprint doi: https://doi.org/10.1101/2023.06.20.545716; this version posted September 19, 2023. The copyright holder for this preprint (which was not certified by peer review) is the author/funder, who has granted bioRxiv a license to display the preprint in perpetuity. It is made available under aCC-BY 4.0 International license.

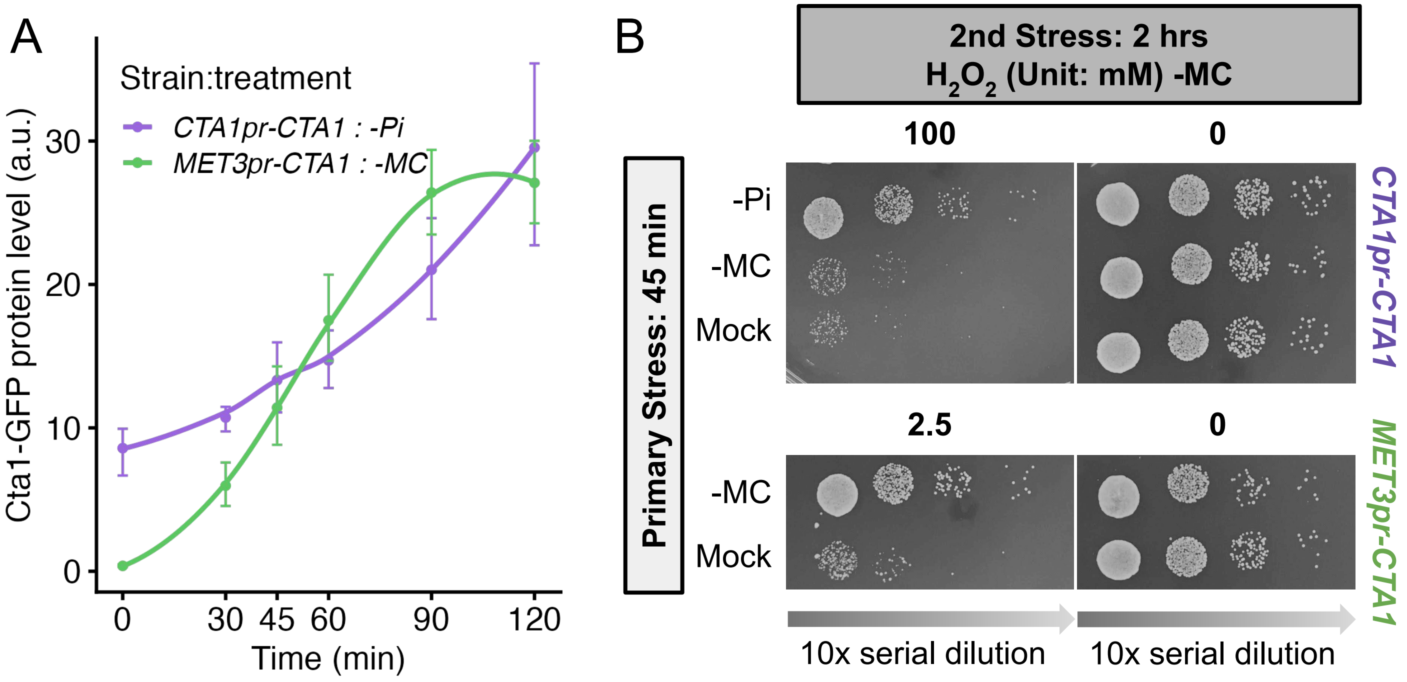

C

Primary stress (45 min)	Secondary Stress (2 hrs)
-Pi : SC w/o Pi	Oxidative: H_2O_2 added to -MC medium
-MC: SC w/o methionine and cysteine	Mock: SC medium
Mock: SC medium	

Supplementary Figure 5. Induction of *CTA1* provides acquired resistance to H_2O_2 in *C. glabrata*. To test if pre-inducing *CTA1* is sufficient to provide ASR for H_2O_2, we replaced the endogenous *CTA1* promoter with the promoter of the *C. glabrata MET3* gene. When grown in SC medium lacking methionine and cysteine (-MC), *CTA1* was induced to a comparable level as in the endogenous *CTA1pr-CTA1* strain under phosphate starvation at 45 minutes (A). Dots represent the mean of at least 3 biological replicates, and the error bars the 95% confidence interval by bootstrapping. The line is the LOESS fit to the data. The endogenous *CTA1pr-CTA1* has a basal expression level that is higher than the *MET3pr-CTA1* (0 min). We also confirmed that the -MC media itself did not provide ASR in the wild type *C. glabrata* cells (B, top). Note that in this set of ASR experiments, all SC medium containing H_2O_2 also lacked methionine and cysteine (C). This allows the *MET3pr-CTA1* to be induced during the secondary stress, mimicking what the wild type strain experiences during the H_2O_2 stress. Using this strain, we found that inducing *CTA1* during the primary stress significantly enhanced the survival of *C. glabrata* cells during the secondary oxidative stress, i.e., providing ASR (B, bottom two rows, -MC vs Mock).

bioRxiv preprint doi: https://doi.org/10.1101/2023.06.20.545716; this version posted September 19, 2023. The copyright holder for this preprint (which was not certified by peer review) is the author/funder, who has granted bioRxiv a license to display the preprint in perpetuity. It is made available under aCC-BY 4.0 International license.

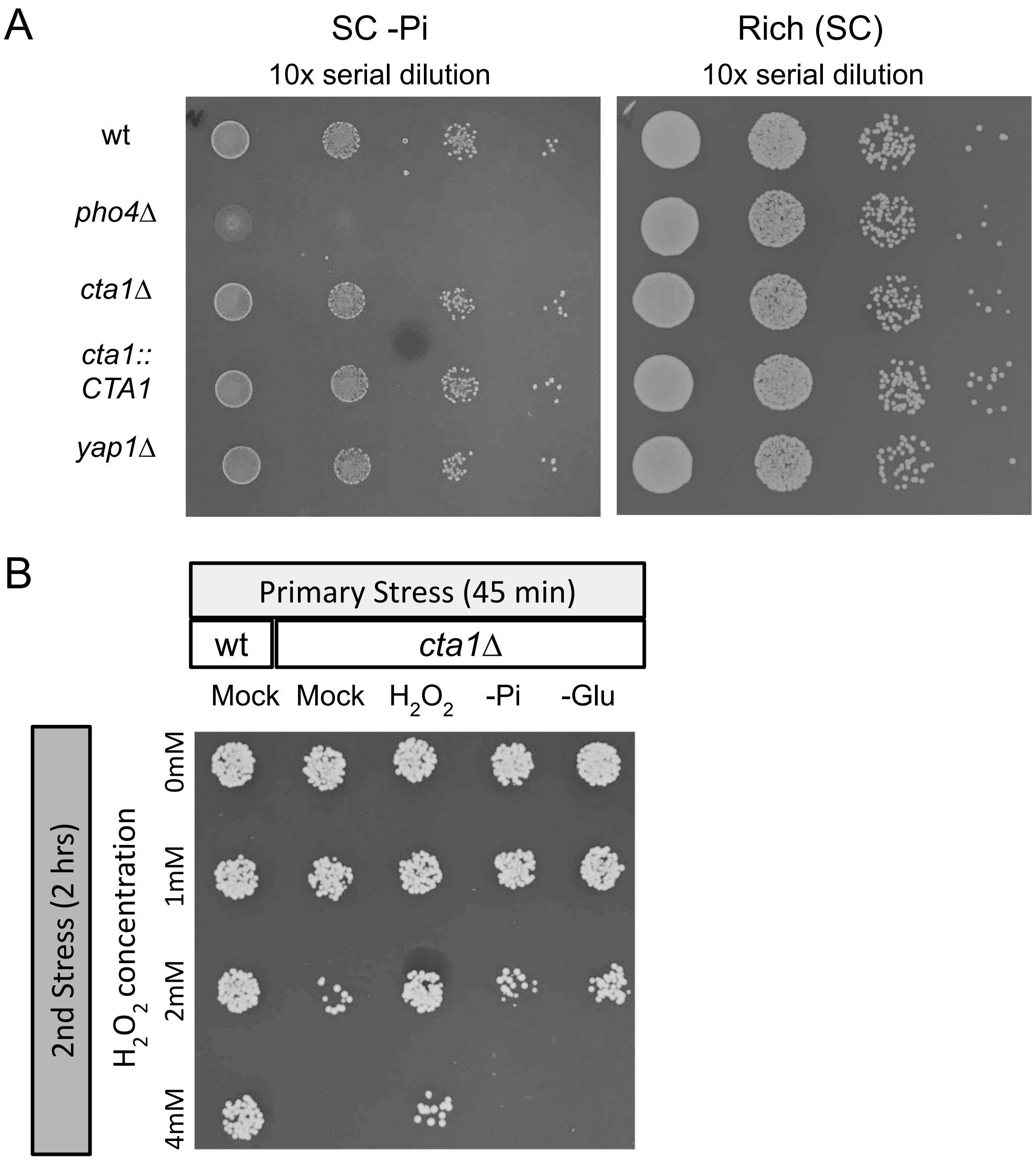

Supplementary Figure 6. Cta1 is not required for survival during the primary phosphate starvation, and is not as important for ASR with mild H_2O_2 used as the primary stress. (A) (Left) Deleting *cta1* and the OSR TF, *yap1*, had no defect on survival under phosphate starvation, while deletion of *pho4*, which is responsible for the PHO response, showed severe growth defects. Mid-log phase cells of the indicated genotypes were spotted on no phosphate SC plates, incubated at 30°C for 48 hours. (Right) Same as (Left) but spotted onto SC plates with 7.5 mM Pi. All images are representatives of >3 biological replicates. (B) *CTA1*'s importance for ASR is dependent on the primary stress type. ASR for wild type and *cta1*Δ were tested with various primary stresses, including phosphate starvation (-Pi), glucose starvation (-Glu, 0.02% glucose), mild H_2O_2 (1.5 mM H_2O_2). ASR experiment was performed as described in the text.

bioRxiv preprint doi: https://doi.org/10.1101/2023.06.20.545716; this version posted September 19, 2023. The copyright holder for this preprint (which was not certified by peer review) is the author/funder, who has granted bioRxiv a license to display the preprint in perpetuity. It is made available under aCC-BY 4.0 International license.

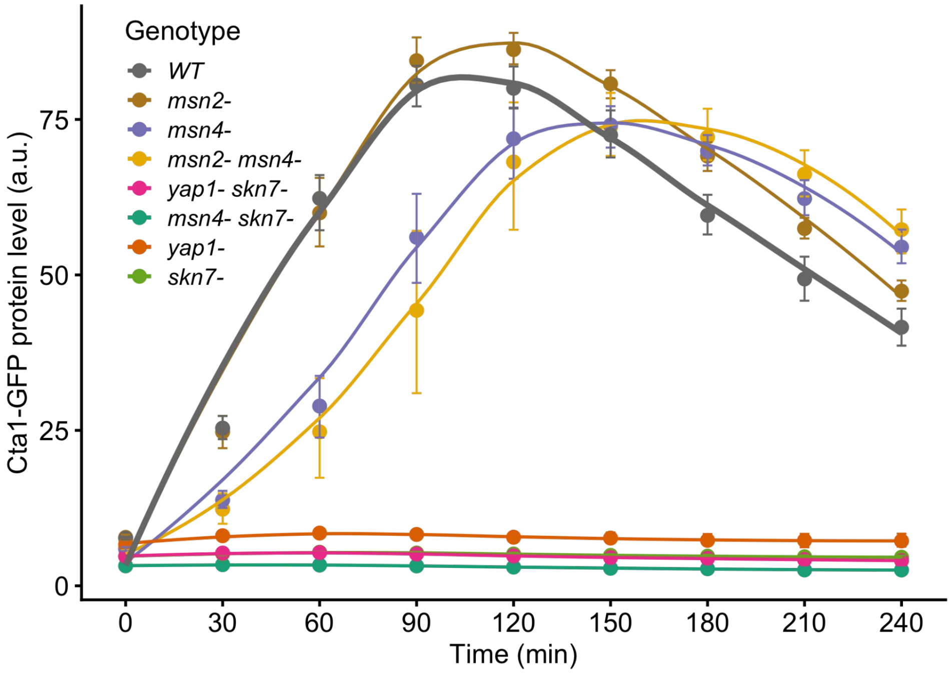

Supplementary Figure 7. TFΔ effects on Cta1-GFP induction under H_2O_2 stress. Cta1-GFP induction under 2 mM H_2O_2 for 4 hours. Yap1, Skn7 play a critical role; Msn4 has a minor contribution while Msn2 is not important. Same features as in Figure 4A.

bioRxiv preprint doi: https://doi.org/10.1101/2023.06.20.545716; this version posted September 19, 2023. The copyright holder for this preprint (which was not certified by peer review) is the author/funder, who has granted bioRxiv a license to display the preprint in perpetuity. It is made available under aCC-BY 4.0 International license.

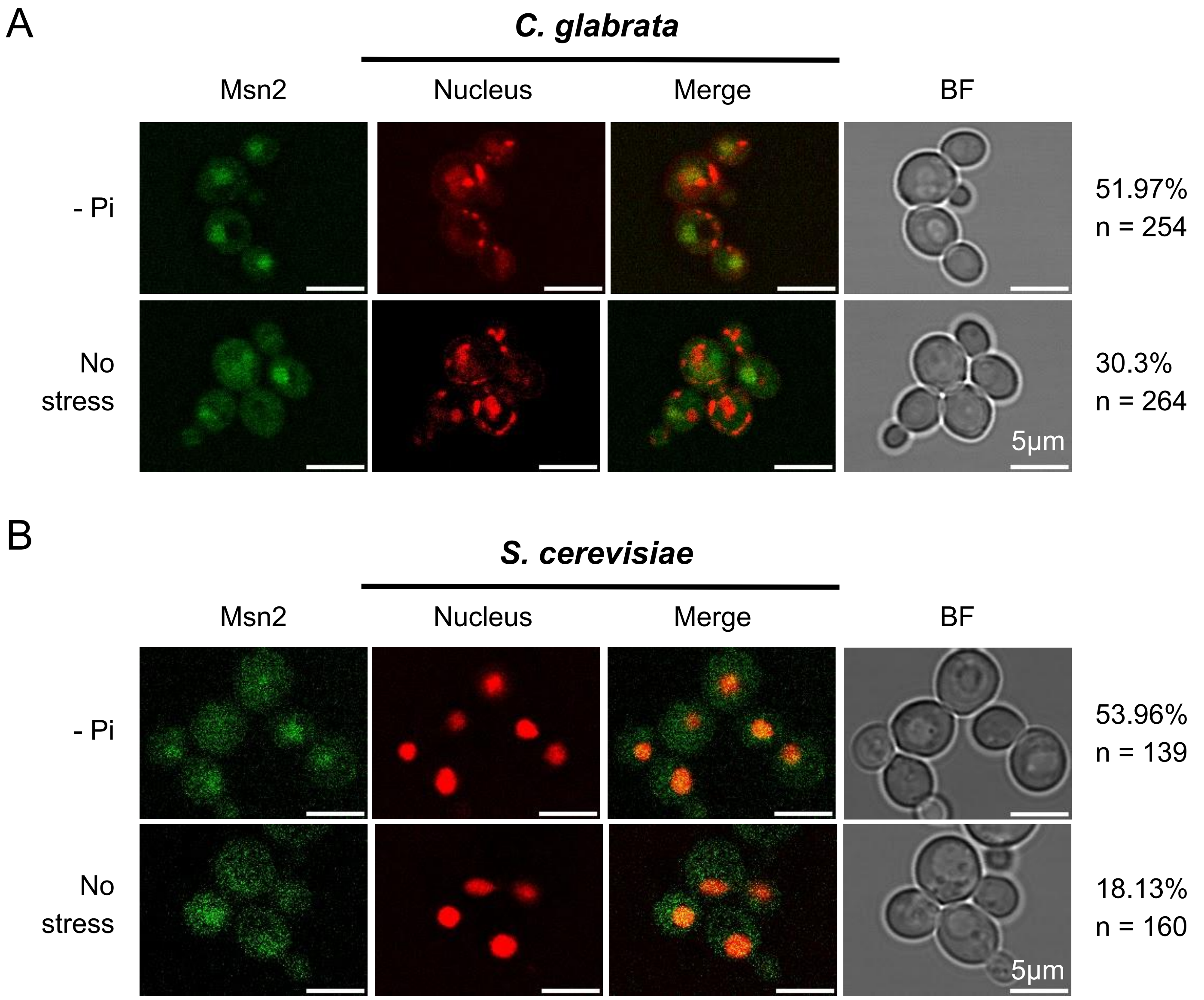

Supplementary Figure 8. *C. glabrata* Msn2 (CgMsn2) and *S. cerevisiae* Msn2 (ScMsn2) translocate into the nucleus upon phosphate starvation. Cellular localization of CgMsn2 (A) and ScMsn2 (B) under phosphate starvation (-Pi) and no stress conditions. All treatments were for 45 minutes. From left to right: i. CgMsn2-yeGFP and ScMsn2-mCherry (pseudo color); ii. nucleus staining with DAPI in *C. glabrata* or Nh6a-iRFP in *S. cerevisiae*; iii. merge of i and ii; iv. bright field; v. percentage of cells with nuclear-localized CgMsn2 or ScMsn2 under each condition (n=number of cells quantified). Scale bars all represent 5 μm in length. DAPI stain more than the nucleus in live *C. glabrata* cells as observed by others (Roetzer *et al.* 2008).

bioRxiv preprint doi: https://doi.org/10.1101/2023.06.20.545716; this version posted September 19, 2023. The copyright holder for this preprint (which was not certified by peer review) is the author/funder, who has granted bioRxiv a license to display the preprint in perpetuity. It is made available under aCC-BY 4.0 International license.

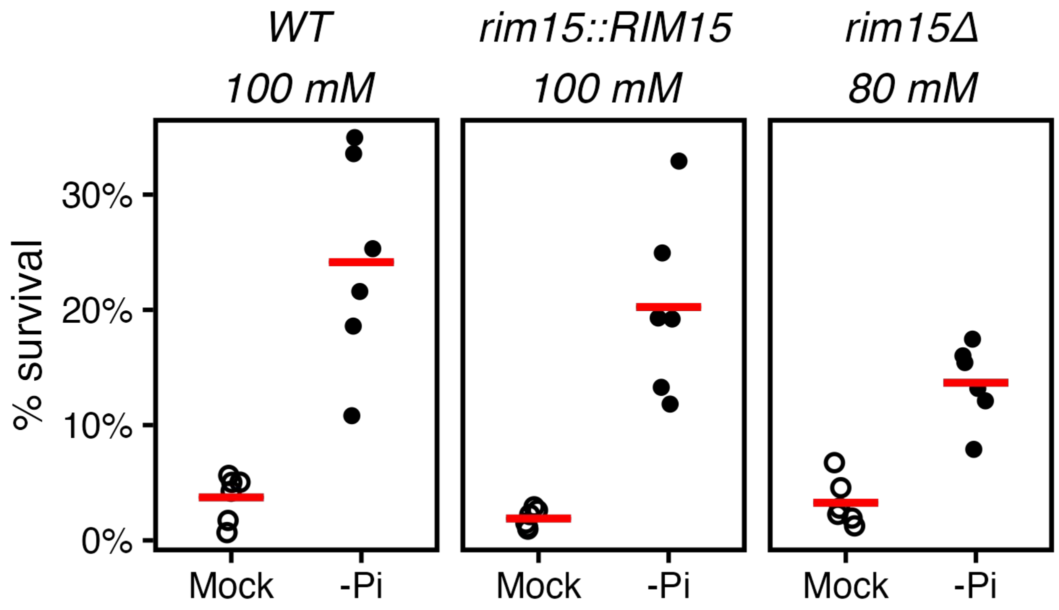

Supplementary Figure 9. *RIM15* complement strain rescues *rim15*Δ ASR defects. We restored *RIM15* at the endogenous locus on the *rim15*Δ background and compared its ASR effect to that in the wild type and *rim15*Δ strains. Concentrations of H_2O_2 were calibrated to achieve a similar basal survival rates (open circles, Kruskal-Wallis rank sum test among the three groups P = 0.27). ASR-scores for the three strains are (95% CI and Wilcoxon signed-rank test P-values in the parenthesis): wild type 9.5 ([5.3, 16.9], P=0.048); *rim15::RIM15* 11.6 ([8.6, 15.2], P=0.048); *rim15*Δ 5.3 ([3.4, 7.5], P=0.048). Based on Mann-Whitney U test, the ASR-score is not significantly different between either *rim15*Δ and wild type, or *rim15::RIM15* and wild type (Holm-Bonferroni-corrected P-values = 0.6 and 0.3, respectively). *rim15*Δ has relatively weak effects on ASR (Fig. 6D) and the wild type's survival rates with primary stress has relatively large variance in this experiment. Both could result in the lack of statistical significance in the comparison between *rim15*Δ and wild type. Instead, the difference in ASR-score between *rim15*Δ and *rim15::RIM15* has a P-value of 0.045. Combined, we conclude that *rim15*Δ reduces ASR and the complement strain rescues the defect.

bioRxiv preprint doi: https://doi.org/10.1101/2023.06.20.545716; this version posted September 19, 2023. The copyright holder for this preprint (which was not certified by peer review) is the author/funder, who has granted bioRxiv a license to display the preprint in perpetuity. It is made available under aCC-BY 4.0 International license.

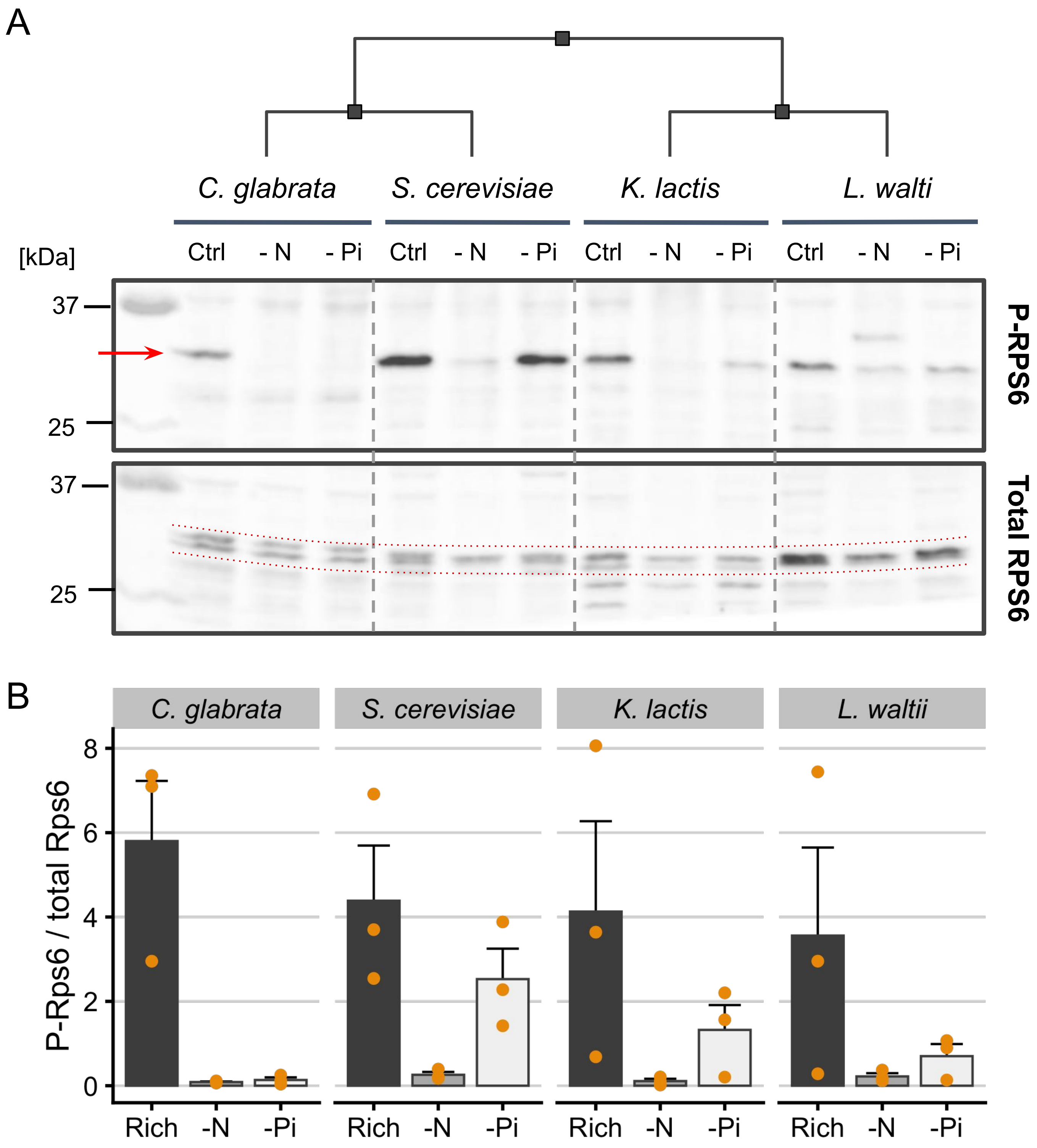

Supplementary Figure 10. TORC1 inhibition by phosphate and nitrogen starvation in diverse yeasts. (A) Western blot for P-Rps6 (top) and total Rps6 (bottom) in log phase *C. glabrata, S. cerevisiae, K. lactis* and *L. walti* cells, grown in rich, nitrogen starvation (-N) and phosphate starvation (-Pi) media for 1 hr at 30 °C. This blot is representative of three biological replicates. The subset of the image including *S. cerevisiae* and *C. glabrata* was already shown in Fig. 7A. The cladogram on the top depicts the phylogeny between the yeasts; the arrow on the top blot indicates the band for the P-Rps6; the two dotted lines in the bottom blot indicate the bands for total Rps6 used for quantitative analysis. (B) Quantification of the ratio of P-Rps6 to total Rps6 based on three replicates. While the trend was obvious, the small sample size (3) limited the power of a paired Student's t-test used to compare the starvation conditions to the rich condition in each species, which resulted in *P*-values that were not significant at a 0.05 level after Bonferroni correction.

bioRxiv preprint doi: https://doi.org/10.1101/2023.06.20.545716; this version posted September 19, 2023. The copyright holder for this preprint (which was not certified by peer review) is the author/funder, who has granted bioRxiv a license to display the preprint in perpetuity. It is made available under aCC-BY 4.0 International license.

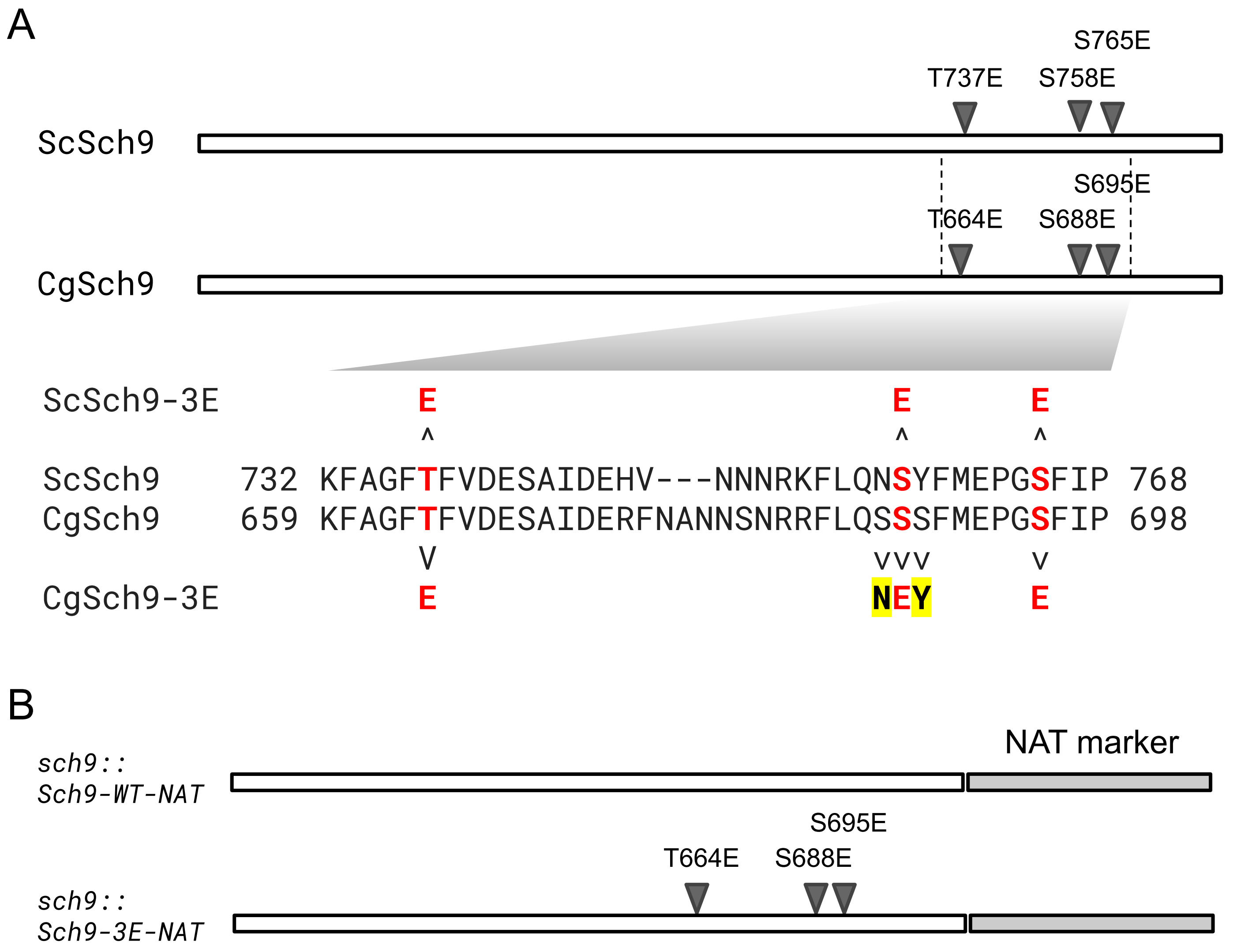

Supplementary Figure 11. Sch9-3E phosphomimetic mutant in *C. glabrata*. (A) Schematic alignment of Sch9 protein in *S. cerevisiae* and *C. glabrata*. The three TORC1 targeted S/T sites in ScSch9, based on Urban *et al.* 2007, and the corresponding sites in the orthologous CgSch9 based on the alignment were labeled with a triangle and the mutations were labeled on the top. The region containing the three S/T sites were shown below as a pairwise sequence alignment below. The S/T sites were shown in red bold fonts. For the second site, S758 (in ScSch9), the corresponding site in CgSch9 are flanked by two serines, making it difficult to determine whether and which serine may be the authentic phosphorylation site. We therefore decided to make three mutations to turn the site into the same sequence as in the ScSCH9-3E mutant. (B) To replace the endogenous *SCH9* gene in *C. glabrata* with the mutant alleles, we added an antibiotic NAT marker at the end of either the wild type *CgSCH9* gene or the *CgSCH9-3E* allele and performed allele swaps. The first construct provides a control for the effect of disrupting the 3' UTR by adding the NAT marker.

bioRxiv preprint doi: https://doi.org/10.1101/2023.06.20.545716; this version posted September 19, 2023. The copyright holder for this preprint (which was not certified by peer review) is the author/funder, who has granted bioRxiv a license to display the preprint in perpetuity. It is made available under aCC-BY 4.0 International license.

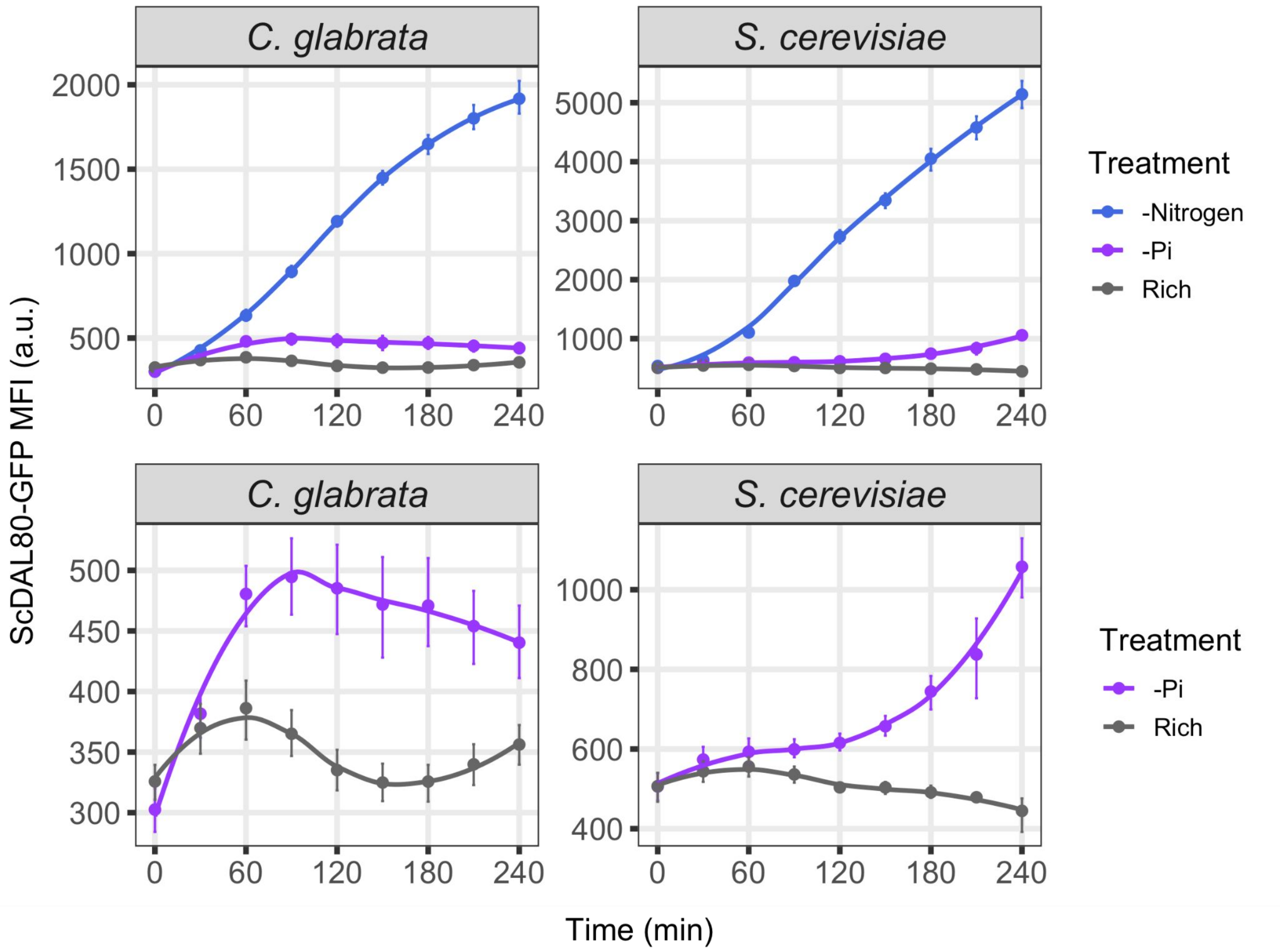

Supplementary Figure 12. *DAL80* induction kinetics under nitrogen and phosphate starvation in *S. cerevisiae* and *C. glabrata*. *ScDAL80pr*-GFP expression levels were monitored via flow cytometry over a time course of 4 hours during nitrogen (-N) or phosphate starvation (-Pi) and rich medium conditions. Dots represent the mean of Median Fluorescent Intensity from > 3 biological replicates; the error bars represent the 95% CI based on 1000 bootstraps. The lines are LOESS fit to the means. The top row shows all three conditions while the bottom row shows the same data without -N to better visualize the induction under -Pi.

bioRxiv preprint doi: https://doi.org/10.1101/2023.06.20.545716; this version posted September 19, 2023. The copyright holder for this preprint (which was not certified by peer review) is the author/funder, who has granted bioRxiv a license to display the preprint in perpetuity. It is made available under aCC-BY 4.0 International license.

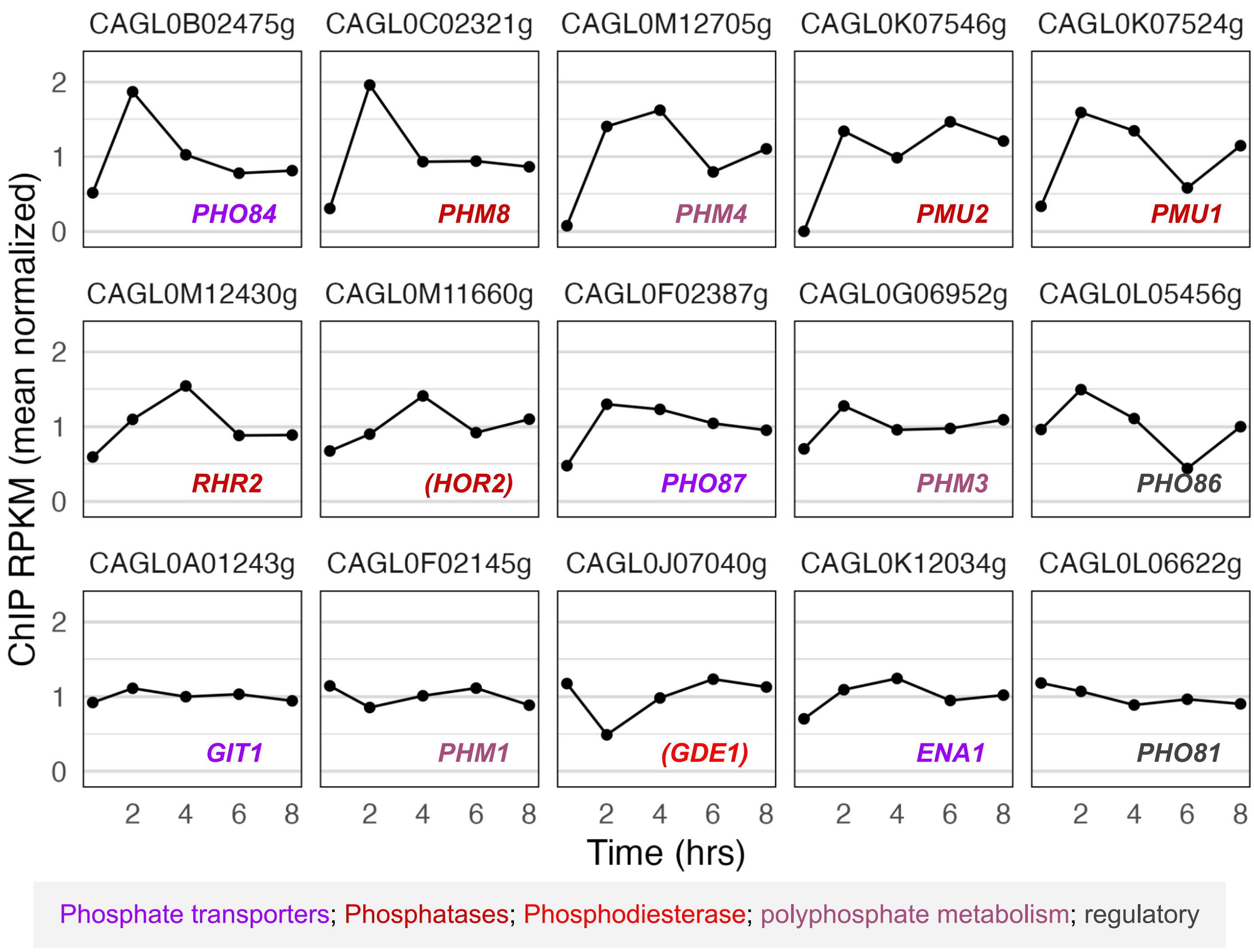

Supplementary Figure 13. Phosphate starvation response is partially induced in *C. glabrata* after the yeast is engulfed by human macrophages. Shown are the phosphate homeostasis genes induced during phosphate starvation in *C. glabrata*. Gene list is from (He *et al.* 2017). PolII ChIP-seq time course data are from (Rai *et al.* 2021), sampled at 0.5h, 2h, 4h, 6h and 8h post infection. For each gene, the ChIP-seq RPKM values were divided by the mean such that the normalized values represent fold changes over the mean for that gene. Systematic gene IDs were labeled on the top of each panel. Gene names were shown inside the panel (names in parentheses were based on *S. cerevisiae* homologs). Colors represented gene functional categories as explained below.

bioRxiv preprint doi: https://doi.org/10.1101/2023.06.20.545716; this version posted September 19, 2023. The copyright holder for this preprint (which was not certified by peer review) is the author/funder, who has granted bioRxiv a license to display the preprint in perpetuity. It is made available under aCC-BY 4.0 International license.

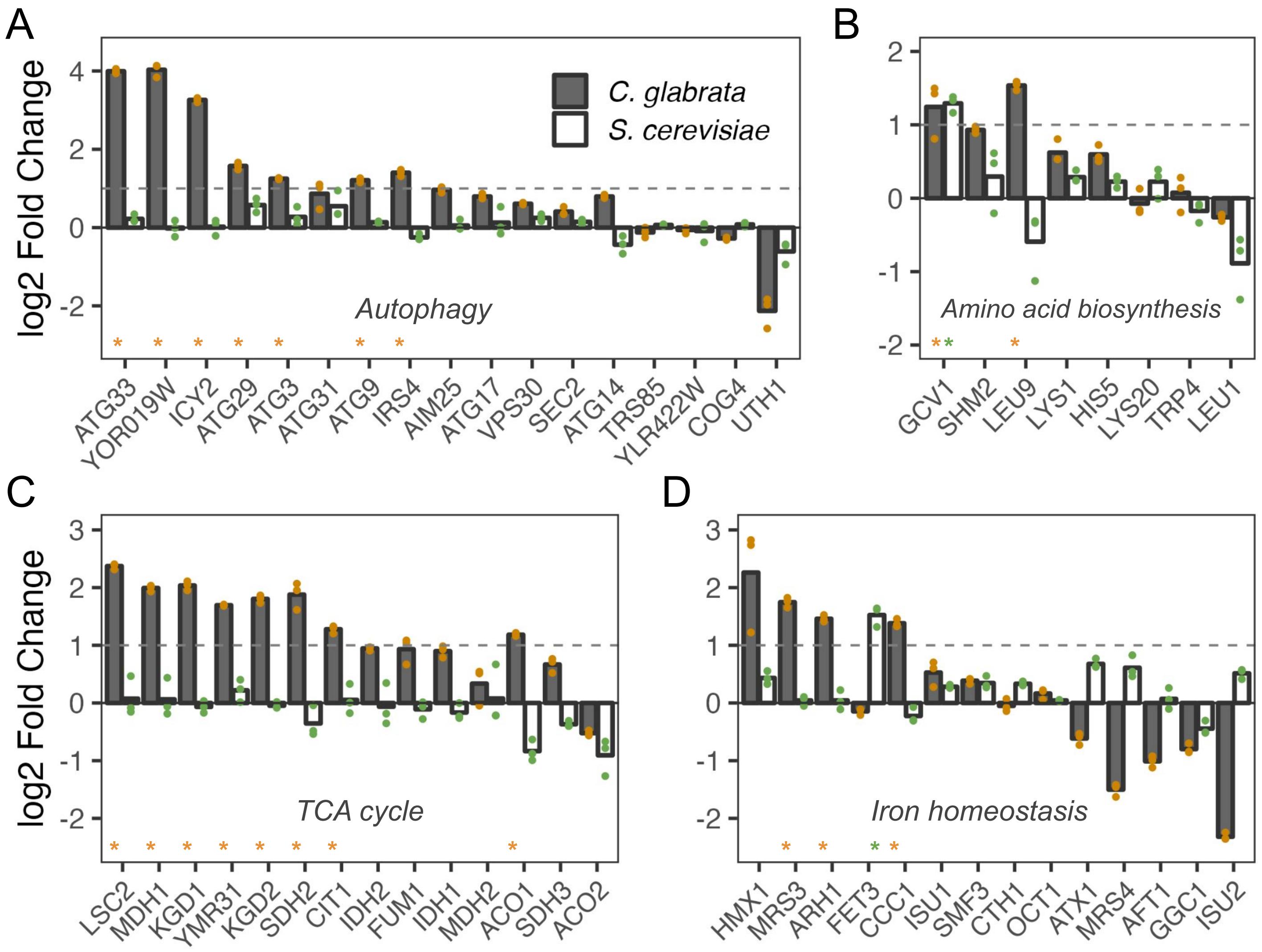

Supplementary figure 14. Genes induced after being engulfed by macrophages are also induced by a short-term phosphate starvation in *C. glabrata*. Data and plots are similar to Figure 2C-F. Gene sets were based on (Rai *et al.* 2021) supplementary figures 2 and 3 for figure 1. Gene names were based on *S. cerevisiae*. An asterisk meant the gene was significantly induced in the species at an FDR of 0.05.

bioRxiv preprint doi: https://doi.org/10.1101/2023.06.20.545716; this version posted September 19, 2023. The copyright holder for this preprint (which was not certified by peer review) is the author/funder, who has granted bioRxiv a license to display the preprint in perpetuity. It is made available under aCC-BY 4.0 International license.

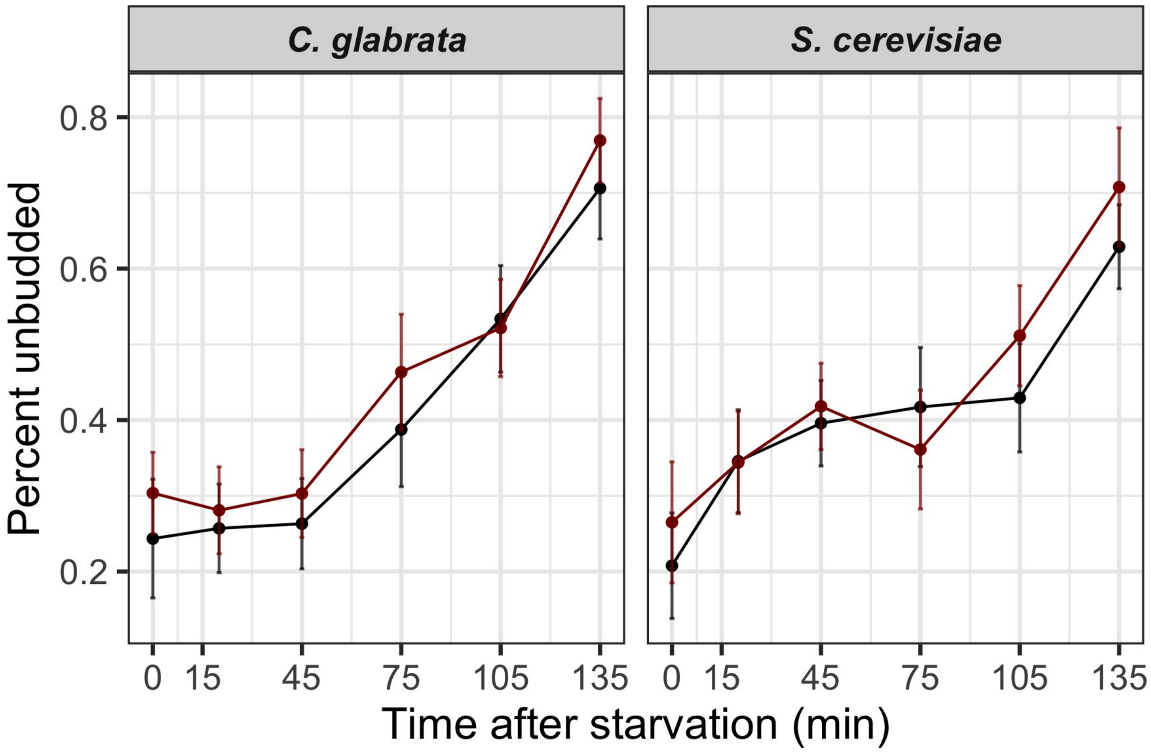

Supplementary Figure 15. Percent unbudded cells during phosphate starvation time course. Cells were taken at 0, 20, 45, 75, 105 and 135 minutes during the time course and assayed for cell morphology by light microscopy (Materials and Methods). The black and dark red dots and lines represent two biological replicates. The dots are the percentage of unbudded cells from >100 total cells examined. The error bars are the 95% confidence interval for the binomial proportion based on normal approximation (Wald Interval).
